# The Rationale Behind the Design Decisions in an Augmented Reality Mobile eHealth Exergame to Increase Physical Activity for Inactive Older People With Heart Failure

**DOI:** 10.2196/50066

**Published:** 2024-08-21

**Authors:** Aseel Berglund, Leonie Klompstra, Helena Orädd, Johan Fallström, Anna Strömberg, Tiny Jaarsma, Erik Berglund

**Affiliations:** 1 Department of Computer and Information Science, Linköping University, Linköping, Sweden; 2 Department of Health, Medicine and Caring Sciences, Linköping University, Linköping, Sweden; 3 Department of Cardiology, Linköping University, Linköping, Sweden

**Keywords:** sedentary, exercise, exertion, exergames, technology, training, inactivity, eHealth application, heart disease, physical activity

## Abstract

Physical activity is important for everyone to maintain and improve health, especially for people with chronic diseases. Mobile exergaming has the potential to increase physical activity and to specifically reach people with poor activity levels. However, commercial mobile exergames are not specially designed for older people with chronic illnesses such as heart failure. The primary aim of this viewpoint is to describe the underlying reasoning guiding the design choices made in developing a mobile exergame, Heart Farming, tailored specifically for sedentary older people diagnosed with heart failure. The goal of the exergame is to increase physical activity levels by increasing the daily walking duration of patients with heart failure by at least 10 minutes. The rationale guiding the design decisions of the mobile exergame is grounded in the thoughtful integration of gamification strategies tailored for application in cardiovascular care. This integration is achieved through applying gamification components, gamification elements, and gamification principles. The Heart Farming mobile exergame is about helping a farmer take care of and expand a virtual farm, with these activities taking place while the patient walks in the real world. The exergame can be adapted to individual preferences and physical condition regarding where, how, when, and how much to play and walk. The exergame is developed using augmented reality so it can be played both indoors and outdoors. Augmented reality technology is used to track the patients’ movement in the real world and to interpret that movement into events in the exergame rather than to augment the mobile user interface.

## Introduction

### Background

Physical activity is important for everyone, especially for people with chronic disease [1]. Low levels of physical activity are commonly reported by patients with cardiac disease, which raises the risk of both illness progression and death [2-4]. Patients with heart failure benefit from physical exercise, but the activity must be tailored to the patient’s baseline condition and the intensity of their symptoms [5]. A promising, innovative way to motivate people to become more physically active is the use of exergames. Exergames are games that combine gameplay with physical activity, requiring people to move in the real world [6]. They lead to an increase in physical activity [7] and have the potential to reach people with poor activity levels [8]. Nintendo Wii and the X-Box Kinect are well-known commercial exergames that have been tested for effectiveness in older people and for patients in cardiac rehabilitation programs [9-11]. Although some exergame studies show benefits for physical activity, a meta-analysis showed that exergaming, when compared to conventional cardiac rehabilitation programs, showed equal effects in terms of improving exercise capacity, quality of life, and mental health [11]. Nevertheless, commercially available exergames, using a “one size fits all” approach, fail to cater adequately to the specific requirements of the older adult population in general [12,13], and more specifically, to individuals dealing with heart failure [14]. A randomized controlled trial using a commercially available, off-the-shelf product demonstrated ineffectiveness in enhancing submaximal exercise capacity among patients with heart failure [15]. Based on interview studies regarding mostly commercial exergames, adult players perceived barriers to exergaming that might be related to the fact that the exergames were initially designed for children or young adults [14,16,17]. Barriers that could hinder older peoples’ abilities to exergame were poor vision, physical function limitations, and other health conditions [14,17].

For this reason, the design of exergames for older people should focus on suitability, usability, and accessibility, as this will increase acceptance. An important aspect to consider in designing exergames for older people is that the location of play should be flexible, for example, offering the potential to be played both indoors and outdoors. This can be realized by developing the exergame for a smartphone, instead of a stationary computer. Another benefit is that smartphones are accessible to the target group. Even though older people might be interested in using mobile phones [18] and mobile health applications can help older people in self-care of illnesses and lifestyle improvement [19], there are no specific mobile exergames designed for older people with heart failure.

The aim of this paper is to describe the principles and technology used in Heart Farming, a mobile exergame designed with the objective of increasing physical activity for inactive older people with heart failure by increasing their walking time by at least 10 minutes each day, as this has been shown to reduce the risk of death or hospitalization by 4% [20]. The Heart Farming exergame was designed and developed using an adapted version of the player-centered, iterative, interdisciplinary, and integrated framework for serious games [21].

### Mobile Exergames for Older People

A previous study shows that outdoor mobile exergames increase older peoples’ engagement, satisfaction, and interest in physical activity [22]. Furthermore, previous research shows that the quality of life for patients with heart conditions can be improved by using mobile phones as health interventions to stimulate physical activities, medication management, dermatology, fall detection, and exercise monitoring [23]. Smartphones are recommended for non–face-to-face physical activity interventions for older people [24]. Smartphones contain built-in technologies, for example, accelerometers, cameras, and sensors, that measure users’ movement and can provide the users with real-time monitoring and feedback on their physical activity without the use of a separate device [25-28]. Furthermore, smartphones provide connectivity, enabling users to get social support that can impact individual motivation positively and consequently improve goal attainment [26,29,30]. Flowie is one of the first mobile exergames to promote increased physical activity among older people and is designed to motivate them to enhance levels of physical activity using a virtual coach [31]. During an 11-day exploratory intervention, participants expressed positive feedback regarding the virtual coach, highlighting increased motivation to engage in exercise. However, quantitative data did not demonstrate a timely increase in physical activity, possibly due to the prototype’s limited activity-sensing capabilities and varying weather conditions during the study. Another mobile exergame is Solitaire Fitness, an asynchronous exergame designed to enhance cognitive and physical ability for older people [32]. The exergame is built on a well-established card game, solitaire, since familiarity can impact the experience positively. The exergame gathers gameplay data so researchers and health professionals can assess project success by measuring user exercise levels and the frequency of features used in the exergame. The authors argue that the general attractiveness and engagement of the game might be improved, while offering health advantages, if new mechanisms are included while keeping it accessible and familiar.

To provide older people with an exergame experience that is perceived as meaningful play, it is important to understand their needs and why they would want to play the exergame [33]. Exergames need to be both attractive to the players as well as effective as an exercise [34]. There are several guidelines for game design [35], exergame design [14,36], and fitness applications [37], as well as motivational factors for mobile games for older adult users [38], all of which need to be considered when designing exergames for older people with heart failure. For example, the benefits gained from playing the exergame need to be clear since they will impact older adults’ use of the exergame [38]. Furthermore, the exergame topic should be adjusted to the interests of older people [36,39,40], and the players’ physical condition should be considered with a suitable difficulty setting [36,39]. Furthermore, the user interface should be adapted to older people [36,39] by, for example, having an easy-to-use user interface and avoiding small objects by, for example, using bigger game characters [36]. Feedback should be both auditory and visual [36], and no feedback should be provided about unachieved goals or unperformed activities, since only positive reinforcement should be used to improve behaviors in exergames for older people [40].

### Gamification Features in Exergames

#### Overview

Gamification can be used to enhance user motivation and engagement with systems [41] by using game design to create gamified experiences [42]. Therefore, gamification features can be integrated in exergames [43]. Matallaoui et al [43] consider exergames to consist of 3 gamification components [41,42] that can be applied for people with heart failure together with gamification principles and gamification elements [44].

#### Gamification Components

**Figure 1.**
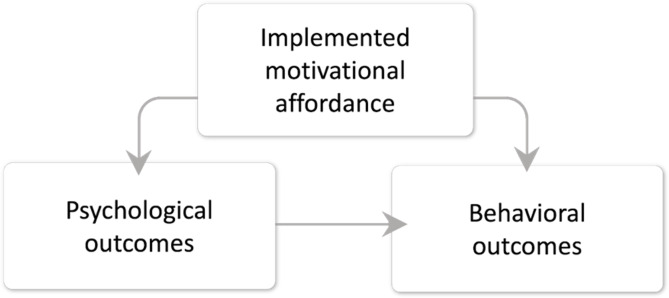
Gamification components adapted from Berglund et al [44], which is published under Creative Commons Attribution 4.0 International License [45].

Gamification can be presented as a process with 3 main components, and these components are the fundamental building blocks that make up a gamified experience [41,44] (Figure 1):

1. *Implemented motivational affordance*: Objects in the user interface of the exergame have properties that allow the players to experience the satisfaction of their psychological, internal, motivational needs. These are used to motivate users to engage with a gamified experience and provide users with a sense of challenge, accomplishment, and reward.
2. *Psychological outcomes*: These are the psychological experiences promoted by the exergame to motivate the user to perform activities. These are the emotional or cognitive effects that gamification can have on users. Examples of psychological outcomes include increased engagement and a sense of accomplishment.
3. *Behavioral outcomes*: These are the behaviors that the exergame will change and that will affect the player’s health. These are the specific actions and behaviors that users take as a result of engaging with a gamified experience. Examples of behavioral outcomes include increased participation, the completion of tasks, and adherence to a desired behavior.

The gamification process starts with motivational affordances that facilitate the psychological experiences that will, together with the implemented affordances, in turn influence the behavioral outcomes (ie, the physical activity for exergames).

#### Gamification Principles

*Gamification principles* are the underlying concepts and strategies that guide the design and implementation of gamification. These principles are the fundamental rules that govern how a game is played and are used to create and extend the player experience [46] and increase overall enjoyment. There are 5 principles that impact the gamification process [47]:

1. *Meaningful purpose*: The goal of the game should align with the users’ motivations [47].
2. *Meaningful choice*: Players have agency over how they achieve their goals and make progress in the game [47] to satisfy their motivational needs [48].
3. *Supporting player archetypes*: The game’s mechanics allow for individual play styles and characteristics [47].
4. *Feedback*: Feedback on how actions performed in the game affect the player’s progress is clearly communicated to the player [47]. Instant feedback makes the game responsive and helps maintain the game’s appeal [49]. Accumulated feedback provides information about the progress of players, which makes it suitable for comparison [49] and can be a source of motivation when players evaluate how close their current behavior is in relation to the goal, thus facilitating self-monitoring [50].
5. *Visibility*: The player can see the status of their current progress, including how much remains to meet their goals or finish a task [47].

#### Gamification Elements

*Gamification elements* are the specific parts that make up the game. The main difference between gamification principles and gamification elements is that gamification principles define how a game works, are used to create an engaging and fun gameplay experience for players, and refer to the overarching design concepts that guide the creation of a game, whereas gamification elements are the specific implementations used to achieve the principles. The 6 most common gamification elements used in health care are as follows [51]:

1. *Leaderboards*: Leaderboards (or high score lists) present the rank of players based on their experience points [51]. Leaderboards enable players to compare themselves with others and evaluate their performance [52].
2. *Levels of achievement or rank*: These are sums of points or credits associated with levels. Levels could evoke a feeling of progression [51].
3. *Digital rewards*: Rewards for achievements in the game can have a positive impact on interest and enjoyment [53] and influence the player’s behavior in different ways [54]. There are 8 forms of rewards [49]:
  - 3.1. *Score systems*: Numbers are used to mark player performance.
  - 3.2. *Experience points reward systems*: Experience points are earned during gameplay and lead to “leveling up” when specified goals are achieved.
  - 3.3. *Item-granting system rewards*: These are virtual items that can be used by players.
  - 3.4. *Resources*: Virtual resources can be collected and used in the game in a manner that affects gameplay.
  - 3.5. *Achievement systems*: Players can collect achievements by fulfilling clearly stated conditions.
  - 3.6. *Feedback messages for immediate rewards*: Some examples include text, sound effects, and animations.
  - 3.7. *Plot animations and pictures*: These are provided after important events in the game such as clearing a new level.
  - 3.8. *Unlocking mechanisms*: Players are rewarded by getting access to game content, for example, new levels achieved when the required experience points are gained.
4. *Real-world prizes*: Players can exchange credits or points earned in the exergame for real-world prizes such as vouchers, goods, or services.
5. *Competitions*: Competitions can be invoked through comparisons with oneself or with peers [50]. Competition appeals to the player’s social needs, which impacts their intrinsic motivation [48].
6. *Social or peer pressure*: Cooperation appeals primarily to the player’s psychological need for relatedness [48] and can complement competition [50].

### Exergames and Augmented Reality

Augmented reality (AR) is a technology that involves the use of sensors and cameras to collect and interpret data from the surrounding environment. AR combines virtual information with the real world and can be used for intelligent displays, 3D registration technology, or intelligent interaction technology [55]. AR technology is usually used to present information (eg, graphics, video, or sound) in a dynamic and interactive manner by overlaying digital content onto the real-world environment [56], and it is highly recommended for health applications that bridge the virtual and real worlds [57]. Furthermore, AR has been used for indoor navigation [28,58,59] by using the technology to determine the user’s position in the physical environment. AR can support localization, but for the technology to work properly, users need to hold the smartphone device in a specific orientation and position [59].

The application of AR in fitness is a novel approach for patient intervention in clinical environments, promotes physical activity, and protects the public from noncommunicable illnesses [60]. Furthermore, AR has the potential to play a role in encouraging and sustaining an active lifestyle, fostering overall well-being among the older adult population [61]. Since AR mobile exergames have the potential to enhance cardiovascular health and physical exercise, they are appropriate for person-centered care interventions [62]. AR technologies have the potential to complement physical interventions in altering human behaviors and influencing physical activity [63]. However, there are several challenges to successfully implement AR techniques in exergames for older people, and usability is one of the challenges. According to a study by Stamm et al [60], older people found it challenging to use complex gestures and navigate menus in AR exergames. Another challenge is safety, and the danger of falling has been identified as a safety challenge that impacts patients’ use of AR exergames [60]. The acceptance of technology is another challenge that needs to be considered in AR exergames [60,64].

To create exergames that are fun to play, the physical environment needs to be considered, since it influences physical activity [65] and the appropriate technology that needs to be used. AR has been used for both outdoor [66] and indoor [67] exergaming. GPS, a satellite-based radio navigation system, is another technology used in many mobile location-based exergames intended for outdoor use [8,68,69]. The error margin of most mobile GPS trackers is in the 5‐ to 10-m range, which means registering shorter distances when walking back and forth (as people playing in smaller areas might have to do) is not possible. However, GPS has a reduced signal strength or lack of signals in indoors environments due to the fact that physical obstructions such as buildings may weaken and even block GPS signals [70,71]. Therefore, GPS is more suitable for outdoors positioning while AR can be used both indoors [72,73] and outdoors [74]. Using the camera for movement tracking results in spatial movement detection nearly 10 times more precise than that achieved by a GPS sensor [58]. AR and GPS can be combined in exergaming. One of the most well-known mobile exergames using both GPS and AR is Pokémon GO, which was shown to increase physical activity [8,68]. Pokémon GO increased user steps by an average of 1473 steps per day over a period of 30 days [8]. However, Pokémon GO has been criticized for the potential harm it could cause via traffic accidents due to players’ distraction or through players placing themselves in dangerous situations or environments while playing [75,76].

In summary, older people with heart failure often lead sedentary lifestyles. AR mobile exergames have the potential to encourage increased physical activity, yet the current lack of mobile exergames designed for both indoor and outdoor use, incorporating gamification features to influence patient behavior, presents a notable gap in the available solutions. In this viewpoint, we describe the rationale behind the design decisions in Heart Farming, a mobile exergame tailored for older people with heart failure.

## Heart Farming: A Mobile Exergame

### Overview

**Figure 2.**
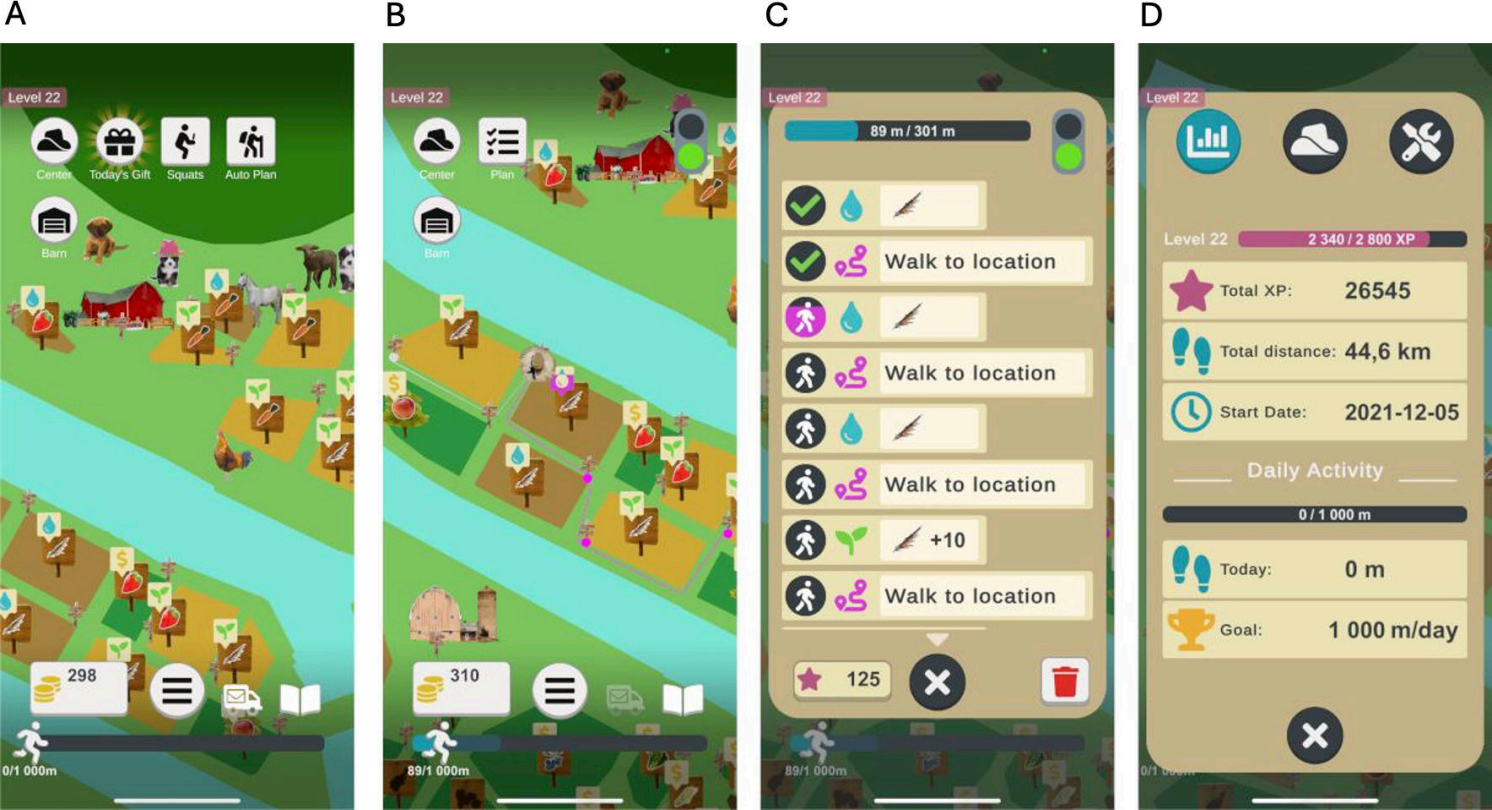
Images from the Heart Farming exergame: (A) main view of the exergame showing the farm before any planning of activities, (B) farm scene as the player plans activities, (C) planned activities are shown in a list view with a progress bar as the player walks, and (D) player statistics and progress in the exergame.

The Heart Farming exergame uses a farming theme, which involves helping a farmer take care of and expand a farm (Figure 2A and B). The players can plan activities in the exergame and then walk in the physical world, indoors or outdoors, to perform the activities they have planned without the need to look at the screen while walking (Figure 2B and C). Available activities are planting and collecting crops of various kinds (eg, sowing carrots, watering strawberries, and harvesting oranges), buying new fields, foraging in the forest, fishing, and selling crops to neighbors on the farm (see Figure 2A-C). Fields and neighbors are visible on a map (Figure 2A and B), and the player must walk in the real world, both to perform the planned activities and to move between locations. As players take care of the farm, they gain experience points, which, over time, unlock new types of crops to farm as well as new neighbors to sell to.

### Gamification Features in Heart Farming

**Figure 3.**
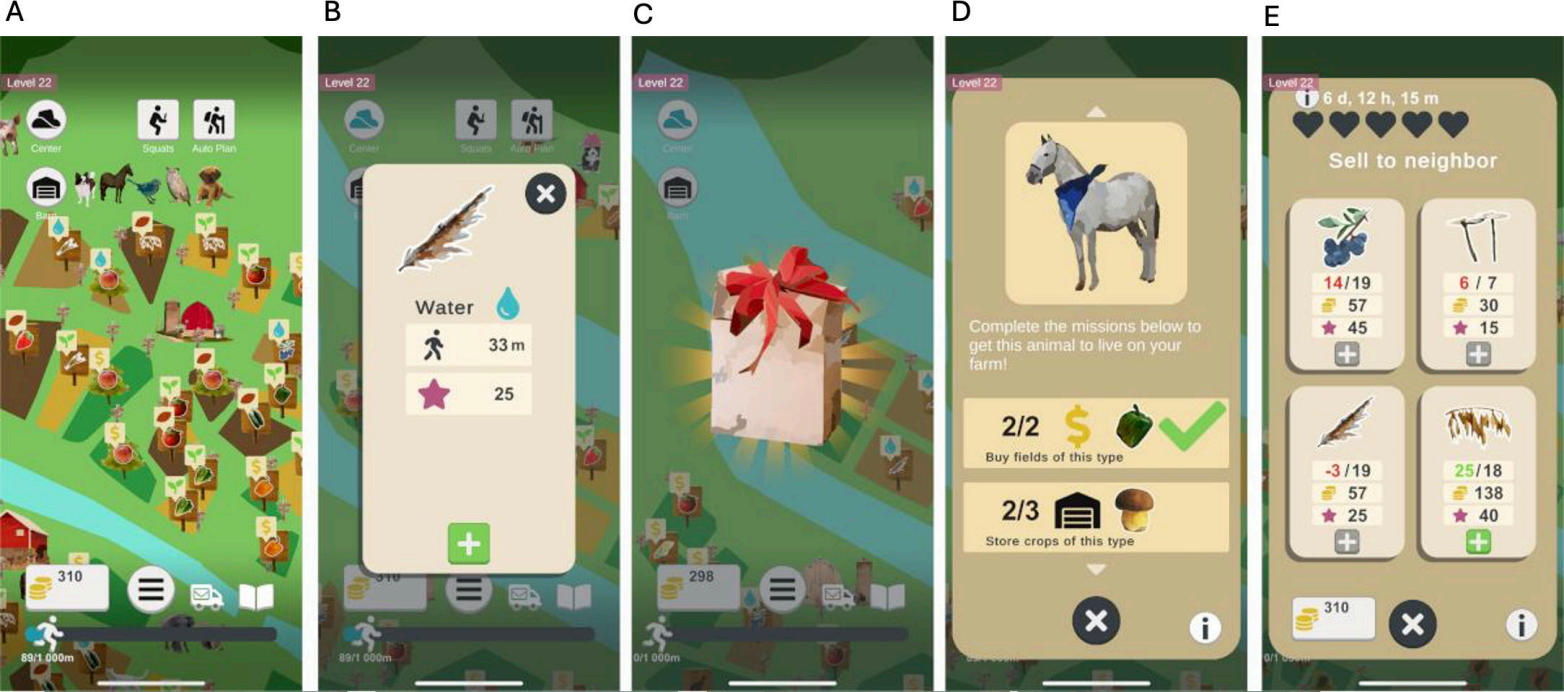
Digital rewards in Heart Farming: (A) score system, (B) experience points, (C) daily gifts, (D) achievement systems, and (E) crops that can be sold to a neighbor.

The gamification components applied in Heart Farming are as follows:

1. *Implemented motivational affordance*: The desired behavior of being physically active is paired with rewards, for example, numeric rewards such as walked distance and experience points. Walked distance builds up to complete the daily goal and is only earned while performing activities in the Heart Farming exergame that require walking. Sums of experience points are associated with levels, which evoke a feeling of progression (Figure 2D). The player gets experience points by performing activities in the exergame, which are based on how many meters of movement the AR system has registered, balanced to match the player’s own physical activity levels. Experience points can be earned from all activities, including sit-to-stand activities, and can be given as a reward, for example, for reaching the daily goal.
2. *Psychological outcomes*: The purpose is to increase players’ motivation and self-efficacy for physical activity, since these factors are important for patients with heart failure [77]. It is common for patients with low exercise capacity to be motivated but lack the confidence to become more physically active [77]. Achieving daily goals in the Heart Farming exergame means that patients are following the physical health recommendation, which might increase their confidence in walking.
3. *Behavioral outcomes*: The goal is to increase physical activity by walking for 10 minutes every day with the Heart Farming exergame. To create a trigger for the player to help create the habit of playing every day, the exergame logs whether players have played or not and sends a reminder at 12 PM if they have not yet played that day. A mission book is also included to give players regular missions; the missions trigger them to do specific activities in the exergame and encourage curiosity about later content (see Figure 3D).

The gamification principles applied in Heart Farming are as follows:

1. *Meaningful purpose*: Real-life physical activity is used to progress in Heart Farming. Players can do 2 types of physical activities: walking in real life and doing sit-to-stand activities. Both activities are performed using the smartphone, which registers the movements and converts them into virtual exergame progress and rewards. Walking is required to meet the player’s daily goal.
2. *Meaningful choice*: Players can choose freely how, when, and where to play. Players who are not interested in optimizing the rewards can choose the automatic mode, which only requires the press of a button before the patient can begin to walk. Players who want more challenges and to perform well in the exergame can instead use the strategic gameplay mode to plan farming activities, which are chosen freely with little or no real analysis of the exergame rules and progress. How players choose to plan their tasks affects the number of rewards they get. Players can also choose to play to complete missions in a mission book. Completing the missions is not obligatory to progress in the exergame, and many tasks in the missions can be completed in more than 1 way to give players choices in how to play the exergame, while others are more specific to encourage players to try new things or extend their farm further. An optional sit-to-stand activity that gives the players experience points or crops is also included to facilitate players with varying degrees of mobility.
3. *Supporting player archetypes*: The goal distance to walk each day is adapted to the players’ exercise capacity. Players are represented in Heart Farming by an avatar in the form of a hat seen from above. As players progress in the exergame, new hats are unlocked, so players can choose a hat that they can identify themselves with. The avatar reflects in-game activities conducted by the player, such as moving on the map when the player walks. Heart Farming is also designed to be adaptable to the players’ exercise capacity. The goal of 10 minutes of walking per day can be based on the number of meters walked during a 6-minute walk test [78]. When players walk their daily goal in the exergame, they receive a trophy. When they have walked twice or 3 times their daily goal, they are rewarded with a smaller experience point boost and a flag animation. After the player has walked 3 times their daily goal, the walking distance is registered and displayed to the player, but in a toned-down fashion without motivational components such as rewards and with the progress bar completely filled.
4. *Feedback*: The players receive multiple types of immediate feedback for performing actions in Heart Farming that communicates clearly how their actions affect progress in the exergame. Instant feedback is provided as visual and auditory information about the behavior that is currently being performed. For example, while walking with a plan in motion, the player can see a progress bar fill up on the current task and hear sounds corresponding to the action being performed (eg, watering sounds while watering a field; see Figure 2C).
5. *Visibility*: All progress in the form of number of meters walked is displayed visually so players can see their overall progress in the Heart Farming exergame as well as their current progress on their daily goal at any time (see Figure 2B-D).

**Figure 4.**
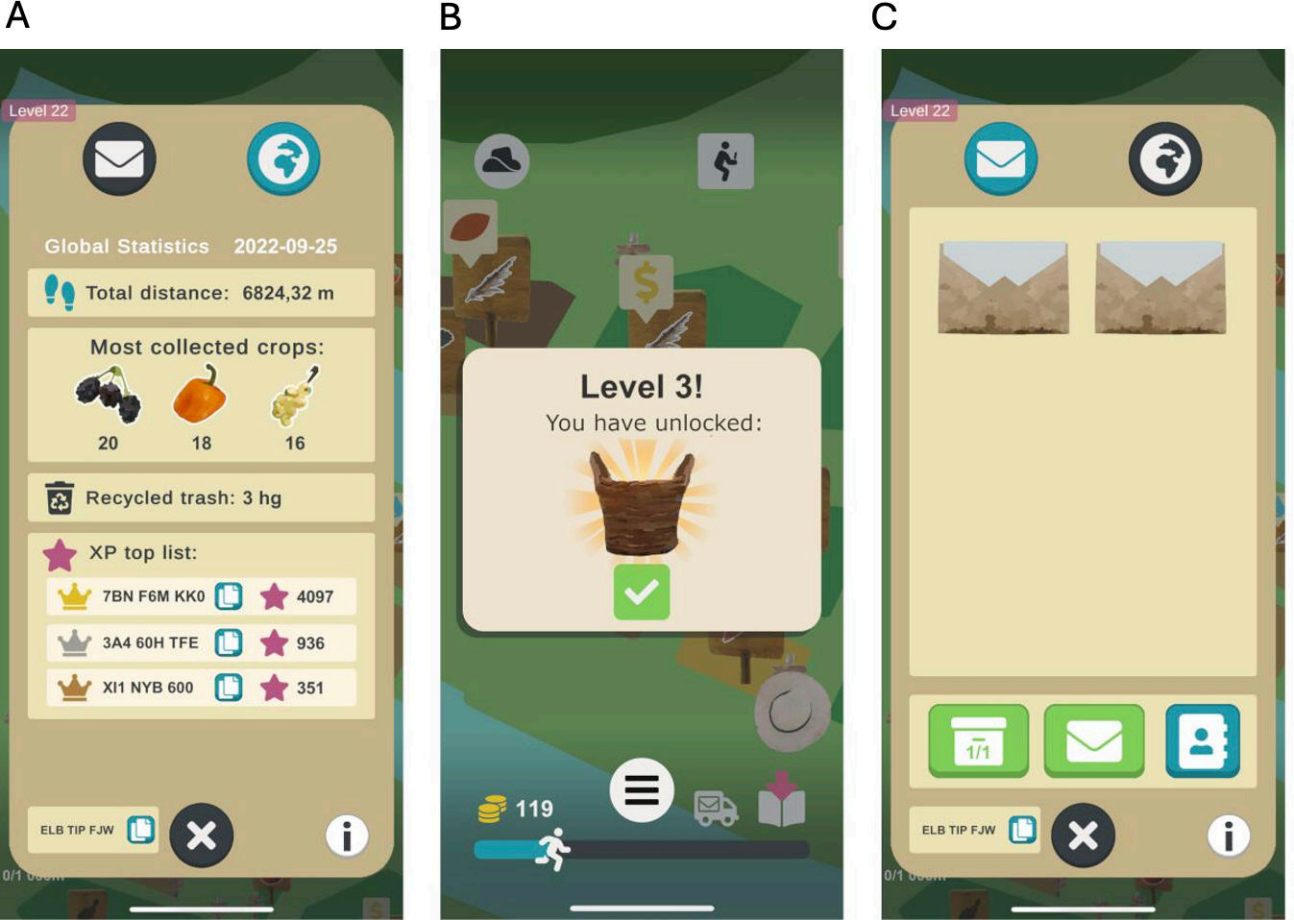
Gamification elements in Heart Farming: (A) leaderboards, (B) levels, and (C) social or peer pressure.

The gamification elements applied in Heart Farming are as follows (Figures3  4):

1. *Leaderboards*: A leaderboard displays the in-game postal codes of the players that gained the most experience points the previous day (Figure 4A). Since low-performing players can be demotivated by a leaderboard when they fall behind and feel that they cannot catch up with others [79], our leaderboard is designed so that players have the same opportunity to be included in it every day, regardless of their performance in the days prior to the current one.
2. *Levels of achievement or rank*: Sums of experience points are associated with levels that evoke a feeling of progression . Each level also unlocks a new type of crop to farm or forage, a new tool that makes farming easier , a new neighbor to whom the player can sell crops (), or a new decorative hat for the farmer to use (Figure 4B). The actual level is always displayed in the upper left corner (Figure 3).
3. *Digital rewards*: The following digital rewards are included in Heart Farming exergame (Figure 3):
  - 3.1. *Score systems*: The total distance walked adds up to reach the daily goal. It is displayed as a progress bar in the lower part of the screen (Figure 3A).
  - 3.2. *Experience points reward systems*: Each activity in the exergame is translated to experience points, which are required for the player to level up over time. A star symbol is used for experience points (Figure 3B).
  - 3.3. *Item-granting system rewards*: Every day, players get a gift of free crops to encourage them to come back to the farm each day (Figure 3C). This incentive fosters a daily habit of playing, which in turn promotes walking every day.
  - 3.4. *Resources*: Coins are needed to buy new fields and are earned from selling crops (Figure 3A).
  - 3.5. *Achievement systems*: Completing specific tasks unlocks unique animals. Each neighbor has hearts that are filled by selling them specific crops, and if all hearts are filled before the end of the week, the player gets a special decoration (Figure 3D).
  - 3.6. *Feedback messages for immediate rewards*: Both animations and sound effects are used to provide feedback when the players perform actions. When players complete the daily goal, they get a reward in the form of an animated trophy and a boost of extra experience points.
  - 3.7. *Plot animations and pictures*: These were not used.
  - 3.8. *Unlocking mechanisms*: The players unlock new tools and crops at higher levels (Figure 4B).
4. *Real-world prizes*: The exergame does not include any real-world prizes.
5. *Competitions*: The exergame does not include any competition.
6. *Social or peer pressure*: Each player has their own in-game postal code that can be used to communicate with other players by sending emojis (eg, smile messages) with positive reinforcement and packages with crops to help each other progress in the exergame (Figure 4C).

### The Exergame Development

**Figure 5.**
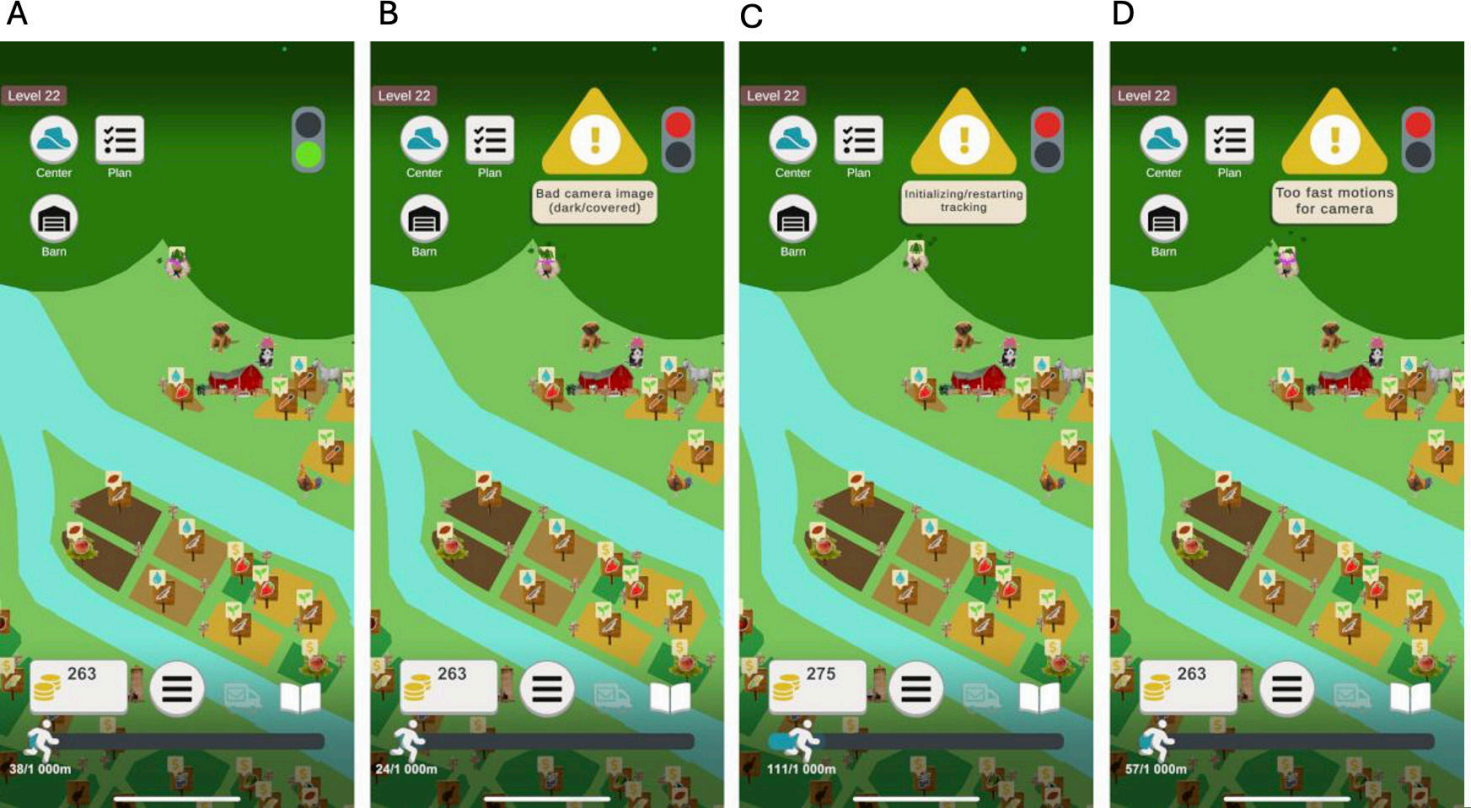
(A) Green traffic light and (B-D) red traffic light with warning messages.

The game development engine Unity was used for the exergame development. The player data are stored in an SQLite database that is connected to a Flask server. The exergame was developed for both the Android and the iOS smartphone operating systems that support AR. The AR is only used to track the players’ movements when walking in the real world. AR enables acceleration-independent tracking by analyzing visual discrepancies among identified landmarks by calculating the change through the AR program’s SLAM solution [80,81]. The status of the AR program is displayed for the player using a stop traffic light with 2 colors: a green light means that the AR can track and register the player’s movement so the player can walk, whereas a red light means that the AR is not working. A red light may either indicate that the AR program is loading or that it is unable to analyze the current camera feed for some reason, for example, it is too dark, the player is moving too fast, or the camera feed is covered. A warning pop-up is displayed for the player and proposes a solution based on the problem. If the camera feed is too dark, the player is prompted to relocate to a brighter place, and if the movement speed is too high (biking or driving speed), the player is prompted with 1 of the 3 different messages: “bad camera image (dark/covered),” “too fast motions for camera,” and “initializing/restarting tracking” (Figure 5).

To mitigate the risk of falling, the player does not need to look at the screen while walking. The player creates a plan with activities and can then walk to perform all activities in the plan without the need to look at the screen. Each activity has a sound to provide feedback for the player about what is happening on the farm.

## Discussion

### Principal Findings

Research indicates that older people with heart failure can potentially reduce the risk of death or hospitalization by 4% simply by adding a daily 10-minute walk to their routine [20]. In response to this, we developed a mobile exergame, Heart Farming, specifically designed to increase their physical activity levels. The rationale that directed the design decisions of the mobile exergame described in this work is based on well-established game design methods to support behavioral changes for inactive patients with heart failure in an enjoyable way. Gamification components, gamification principles, and gamification elements have been applied for designing the exergame. Heart Farming is based on a farming theme, and to play the game, players need to walk in the real world.

Exergames can persuade people to exercise more when they are designed for exercising [82], but defining appropriate goals is difficult, and therefore, official institutional guideline values can be used appropriately as a starting reference point [64]. Since regular physical activity can help preserve health and enhance the quality of life for the patient group, the exergame is designed so that the patient comes back to the exergame and plays every day, for example, using daily gifts with different crops and daily reminders when the patient has not walked.

The gamification components in Heart Farming are provided to increase the patients’ physical activity, which requires behavioral changes. Since motivation drives humans to perform and can arise from physiological or psychological needs, thoughts, or emotions [83], the design and technical implementation is applied to balance the patients’ physical needs with an elusive fun factor. To perform a behavior, a person must be motivated, have sufficient ability, and have a trigger to prompt the behavior [84]. Thus, the design of Heart Farming aims to increase the motivation to engage in physical activity and keep it at a level where the patients’ abilities are sufficient to perform the activity. For the trigger, the exergame sends a reminder at 12 PM if the player has not yet played that day. By setting the reminder around lunch time, the patient can associate playing the exergame with an existing habit of eating lunch, making it easier to create a new habit either before or after eating. According to Stawarz et al [85], reminders can, however, hinder habit formation if the user gets reliant on the reminders, which causes the habit to die out if the user stops using the app. By only sending reminders to players who have not played yet, only those who need the reminder get them, and as they start connecting a trigger event in their lives to the new habit, the reminders might phase out naturally over time. A potential problem with having a set time for the reminders is that the time might not work for some patients, as they might be busy or might not have any trigger events such as having lunch around that time. Allowing patients to set their own reminder time might make it easier for them to form a habit.

The only way for players to customize their avatar is to change the hat that is visible from above. Furthermore, the players only unlock new hats after reaching higher levels and thus have very limited choices in the beginning. This could make it harder to relate to and identify with the avatar, which has been shown to be important for games that aim to change behavior [82]. However, changing to a more human avatar could raise issues regarding how much personalization is needed to ensure that nobody feels alienated by the available choices. It may therefore be better to stick to an avatar where the patient must imagine what the farmer looks like on their own.

To avoid endangering the patients’ health and to avoid physical overexertion, as demonstrated with Pokémon GO [86], players will not be encouraged to walk after reaching 3 times their daily goal, but they are not prohibited from continuing to walk if they wish to exercise further, and the data will still be collected and stored by the game. This allows for individual play styles and characteristics, which has been identified as an important principle [44]. Players can also decide which play style they want to use when playing the exergame (automatic plan or strategic plan).

Players that wish to compare themselves to others as a way of challenging themselves to do better can use the leaderboard to see the scores from other players from the previous day and work toward reaching the top of the board. By resetting scores daily and collecting scores 1 day at a time, new players or those who have not played the exergame in a while still have an opportunity to top the leaderboard. Players can compare their results by looking at the leaderboard or by talking to other players about their scores and progress. Players can also interact with other players in the exergame by sending messages. The possibility to play with others has been identified as a desired feature in exergaming for patients with heart failure [14,17]. The question of whether sending messages is an optimal format for interaction should be further explored.

To not endanger the patients’ health, the AR technique is used to track the players’ movements when walking rather than to enhance the real world. The AR technology is used to detect the players’ motion in the real world so players can choose to be physically active indoors, outdoors, or both. Using AR technology may have some practical constraints. When using the AR as a tool for motion detection, it is important that the players understand why and how the camera in the smartphone is used, so they feel confident when they play and can interact with the exergame as required. For Heart Farming, the players can play the exergame by either holding the smartphone in front of them or hanging the smartphone in a bag around the neck. In both cases, the front-facing camera must have a clear view of the surroundings. However, the player does not need to look at the screen while walking, which mitigates the risk of falling. The players get audio feedback informing them about the performed activities. AR is not the only technique available for tracking the players’ movement. GPS and step counting are 2 possible alternatives, both of which could be used even if the smartphone is kept inside a pocket. Step counting is an offline data stream that is reported occasionally, so it is not directly accessible by the system for instant feedback. Step counting also does not provide good data for users that move slowly or generate little vibration from their steps and is therefore unsuitable for the target patient group. Since GPS lacks precision or fails entirely indoors, it is not suitable for indoor use. However, GPS could be incorporated for outdoor use only, with AR still used for indoor movement, although making the choice between the technologies in an easy way could be challenging.

The effects of playing the Heart Farming exergame will be investigated in a randomized control trial with patients with heart failure, and the data are currently being collected. In the study, the patients’ experiences from playing the exergame will also be investigated.

### Conclusion

Heart Farming is a mobile exergame tailored to increase physical activity in inactive older people with heart failure by encouraging them to walk for at least 10 minutes each day. The Heart Farming exergame is about helping a farmer take care of and expand a virtual farm. The advantages of the proposed exergame can be summarized as follows:

- The Heart Farming exergame detects patients’ physical activities by using AR technology to detect patients’ movements in the real world, both indoors and outdoors.
- The AR technology in the Heart Farming exergame continuously registers patients’ movements in the real world and interprets them into events in the exergame, which then generates both visual and auditory feedback.
- The Heart Farming exergame can support patients’ own individual preferences and conditions regarding where (indoors or outdoors), how (automatic or strategic gameplay), how much (patients are rewarded for once, twice, or 3 times their daily goal, and after that, progress is registered and displayed without rewards), and when (patients are remined at 12 PM if they have not played that day) to play.

## Abbreviations

AR: augmented reality
